# High risk for revision after shoulder arthroplasty for failed osteosynthesis of proximal humeral fractures: A matched pair analysis of 285 cases from the Danish Shoulder Arthroplasty Registry

**DOI:** 10.1080/17453674.2018.1450207

**Published:** 2018-03-14

**Authors:** Marc Randall Kristensen, Jeppe Vejlgaard Rasmussen, Brian Elmengaard, Steen Lund Jensen, Bo Sanderhoff Olsen, Stig Brorson

**Affiliations:** 1 Department of Orthopaedic Surgery, Herlev Hospital, University of Copenhagen, Herlev, Denmark; 2 Department of Orthopaedic Surgery, Aarhus University Hospital, Aarhus, Denmark; 3 Department of Orthopaedic Surgery, Aalborg University Hospital, Aalborg, Denmark; 4 Department of Orthopaedic Surgery, Zealand University Hospital, University of Copenhagen, Køge, Denmark

## Abstract

**Background and purpose:**

It is unclear whether previous osteosynthesis is a risk factor for inferior outcome following shoulder arthroplasty for a proximal humeral fracture. We used data from the Danish Shoulder Arthroplasty Registry (DSR) to examine this question.

**Patients and methods:**

All 285 patients treated with a shoulder arthroplasty after failed osteosynthesis of a proximal humeral fracture reported to DSR from 2006 to 2013 were included. Each case was matched with 2 controls (570) treated with a primary shoulder arthroplasty for an acute proximal humeral fracture. Patient reported outcome was assessed using the Western Ontario Osteoarthritis of the Shoulder index (WOOS) and the relative risk of revision was reported.

**Results:**

The mean WOOS was 46 (SD 25) for a shoulder arthroplasty after failed osteosynthesis and 52 (27) after a primary shoulder arthroplasty. The relative risk of revision for a shoulder arthroplasty after failed osteosynthesis was 2 with a primary arthroplasty for fracture as reference. In a separate analysis of patients treated by locking plate the mean WOOS was 46 (24), with a relative risk of revision at 1.5 with a primary arthroplasty as reference.

**Interpretation:**

Compared with primary arthroplasty for proximal humeral fracture, we found an inferior patient-reported outcome and a substantial risk of revision for patients treated with a shoulder arthroplasty after failed osteosynthesis for a proximal humeral fracture. The risk and burdens of additional surgery should be accounted for when deciding on the primary surgical procedure.

Treatment modalities for a proximal humeral fracture vary from nonsurgical treatment including immobilization and physiotherapy to surgical treatment including operative fixation with head-preserving techniques or a primary shoulder arthroplasty. Locking-plate osteosynthesis has been popular in recent years (Jost et al. 2013, Sun et al. 2013), but many complications and reoperations have been reported (Clavert et al. 2010, Sproul et al. 2011), especially in complex fracture patterns (Brorson et al. 2011, 2012). The most commonly reported complications are avascular necrosis of the humeral head, screw penetration, and glenoid destruction (Spross et al. 2012, Brorson 2013, Jost et al. 2013).

The reported reoperation rate of proximal humeral fractures treated with locking plates varies in the literature, ranging from 3% to 44% (Bjorkenheim et al. 2004, Clavert et al. 2010, Brorson et al. 2011, 2012, Spross et al. 2012). Recently, a large multicenter study found no better outcome after surgery compared with nonsurgical treatment (Rangan et al. 2015).

Shoulder arthroplasty is often offered when revision of a failed osteosynthesis is required. Shoulder arthroplasty entails a risk of complications too, including persistent pain, infection, instability, neurologic injury, tuberosity migration and vanishing, rotator cuff tear, heterotopic ossification, glenoid erosion, stiffness, and periprosthetic fractures, which may eventually lead to revision surgery (Plausinis et al. 2005, Kontakis et al. 2008).

It has been reported that the patient-reported outcome measurements (PROM) after early shoulder arthroplasty are significantly better than after late shoulder arthroplasty (Bosch et al. 1998). However, it has also been reported that improved outcome is achieved following treatment of fracture sequelae by shoulder arthroplasty (Boileau et al. 2001, Jost et al. 2013, Alentorn-Geli et al. 2014). To our knowledge, only studies with a small number of patients have reported whether previous osteosynthesis is a risk factor for an inferior outcome of shoulder arthroplasty for proximal humeral fractures (Hussey et al. 2015, Dezfuli et al. 2016, Grubhofer et al. 2017).

We hypothesized that previous osteosynthesis is a risk factor for inferior outcome following shoulder arthroplasty for a proximal humeral fracture. Thus, we evaluated patient-reported outcome and risk of revision after arthroplasty for proximal humeral fracture.

## Patients and methods

Data were obtained from the Danish Shoulder Arthroplasty Registry (DSR). The DSR was established in 2004 with the purpose of systematically collecting data on all primary and revision arthroplasties on a national level. The registry is independent of commercial interests and financed by the Danish counties. Since 2006, reporting has been mandatory for all Danish hospitals and private clinics performing shoulder arthroplasty surgery and the completeness of reporting has been above 92% (Jensen et al. 2016). Data are reported online by the surgeon at the time of operation. A revision is defined as removal or exchange of the humeral component or the addition of a glenoid component (Rasmussen et al. 2012, 2014). Patients are identified in the register by their unique civil registration number (CPR). Thus, a revision arthroplasty is linked to the primary arthroplasty using the CPR.

Patient-reported data are collected 10–14 months postoperatively for both primary prostheses and revision prostheses by mail, using the Western Ontario Osteoarthritis of the Shoulder (WOOS) index. WOOS is a patient-administrated questionnaire with 19 questions regarding the shoulder-related quality of life, resulting in a percentage score with 100 being the best. To improve the response rate, we sent a single reminder to non-responders and to those returning an incomplete questionnaire. If a patient dies or the shoulder arthroplasty is revised within 1 year postoperatively WOOS cannot be obtained.

All patients reported to DSR from 2006 to 2013 with a shoulder arthroplasty after failed osteosynthesis of a proximal humeral fracture were identified. For comparison, we chose a group of patients with an acute proximal humeral fracture treated with a primary shoulder arthroplasty and reported to the DSR in the same time period (2006–2013). We matched each patient with a shoulder arthroplasty after a failed osteosynthesis with 2 patients treated by a primary shoulder arthroplasty. They were selected to match in terms of sex, age at the time of the arthroplasty operation, and whether the WOOS questionnaire was completed or not, resulting in the same WOOS response rate in the 2 groups. If more than 2 controls matched all three parameters we used those with a date of birth closest to that of the case. For patients younger than 50 or older than 90, the number of eligible patients with a primary shoulder arthroplasty were low. For these patients, we chose to match in age groups (21–30, 31–40, 41–50 and 91–100). 1 patient was reported to DSR with bilateral surgery and included as two independent cases.

### Statistics

Data were analyzed using a chi-square test for binary outcomes and Student’s t-test for continuous outcomes (Lumley et al. 2002). A Cox regression model was used to calculate the relative risk of revision. Sex and age, as a binary variable with 65 years as the threshold, were included in the model. The Kaplan–Meier method was used to illustrate the unadjusted survival rates. SPSS was used for the statistical analysis (IBM Corp, Armonk, NY, USA). The level of statistical significance was set at p < 0.05 and p-values were 2-tailed.

## Ethics, funding, and potential conflicts of interest

The Danish Data Protection Agency (journal number: HEH-2015-008, date of issue February 4, 2015) and the Danish Health Authority (journal number: 3-3013-1075/1/, date of issue August 10, 2015) approved the study. No funding was recieved for this study. No conflicts of interest were declared.

## Results

### Demographics

**Figure 1.**
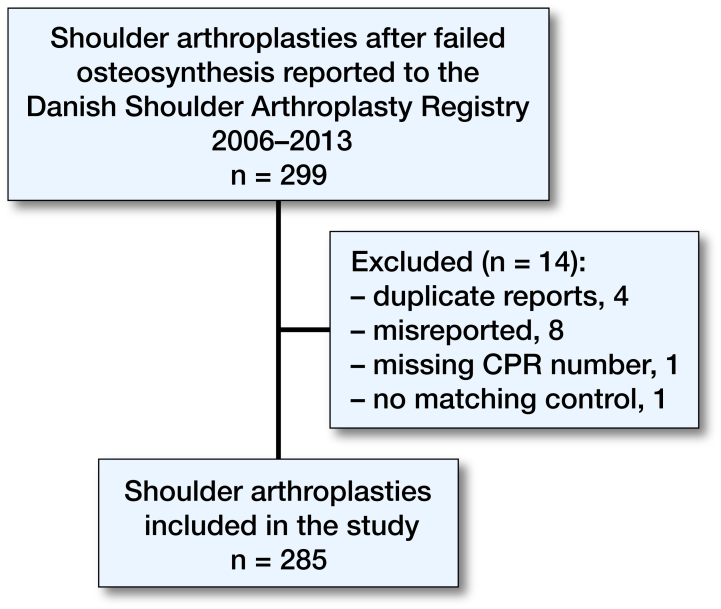
Flowchart of included cases. 299 were reported to the registry, 285 were included in this study.

Between 2006 and 2013, 299 shoulders were reported to DSR as having a shoulder arthroplasty after failed osteosynthesis of a proximal humeral fracture. 14 cases were excluded, leaving 285 cases and 570 controls (Figure 1). The data were extracted from the registry in March 2015.

The follow-up time of the patients was 36 months (SD 27) among the patients with a secondary arthroplasty and 39 months (SD 25) among patients with a primary arthroplasty (p = 0.07).

Mean age of patients with a secondary arthroplasty was 66 years (SD 12) and 213 (75%) patients were women. A locking plate was used in 73% of the patients (Table 1). In 7% of the patients it was not possible to find information regarding the type of osteosynthesis. In 39% of the patients avascular necrosis was registered as the reason for failure leading to shoulder arthroplasty. Fracture displacement (17%) and pseudarthrosis (13%) were other common reasons for revision (Table 2).

**Table 1.** Type of osteosynthesis in 285 cases

	n	%
Locking plate	208	73.0
K-wires	17	6.0
Non-locking plate	15	5.3
Screws only	10	3.5
Intramedullary nail	9	3.2
Helix wire	4	1.4
Intramedullary nail (thin)	3	1.1
Missing	19	6.7

**Table 2.** Mode of osteosynthesis failure and outcome

		Mean	Revisions
Mode	n (%)	WOOS	n (%)
Avascular necrosis	107 (38)	46	9 (29)
Fracture displacement	49 (17)	45	6 (19)
Pseudarthrosis/non-union	36 (13)	49	7 (23)
Secondary arthrosis	12 (4)	46	2 (6)
Infection	9 (3)	32	3 (10)
Screw penetration	9 (3)	51	0 (0)
Pain	8 (3)	32	1 (3)
Rotator cuff tear	6 (2)	48	0 (0)
Implant failure	6 (2)	50	0 (0)
New fracture	2 (1)	41	0 (0)
Missing	41 (14)	47	3 (10)

68% of the patients were treated with a stemmed hemiarthroplasty and 23% with a reverse shoulder arthroplasty. The remaining 9% were reoperated by a resurfacing prosthesis, total anatomical arthroplasty, or a bipolar shoulder arthroplasty. In the group of patients with a primary shoulder arthroplasty 94% were treated with a stemmed hemiarthroplasty. The remaining 6% were treated with a reverse shoulder arthroplasty, resurfacing prosthesis, or total anatomical arthroplasty.

## Patient-reported outcome

21 patients died and 5 patients were revised within 1 year. Of the remaining 259 patients 77% responded to the WOOS questionnaire. The mean WOOS of patients with a shoulder arthroplasty after failed osteosynthesis was 46 (SD 25). The mean WOOS of patients with a primary shoulder arthroplasty was 52 (27) (p = 0.005). The mean WOOS of the 2 groups was similar, except for the patients revised because of pain or infection, who had a mean WOOS score of 32. However, the numbers were too small for meaningful statistical comparison (Table 2). A subanalysis of the results after treatment with the different kind of prostheses in our study gives an average WOOS of 46 for both stemmed hemiarthroplasty and reverse arthroplasty.

## Risk of revision

31 (11%) of the 285 patients with a secondary shoulder arthroplasty after a failed osteosynthesis had a revision of the arthroplasty. The most common reasons for revision arthroplasty were dislocation, rotator cuff tears, and infection (Table 3). In the group of patients treated with a primary shoulder arthroplasty 34 (6%) were revised.

**Table 3.** Causes of revision in cases (secondary arthroplasties) and controls (primary arthroplasties)

	Cases	Controls
Cause	n = 31	n = 34
Dislocation	8	8
Rotator cuff tears	5	10
Infection	5	4
Pain	5	1
Other	2	5
Glenoid erosion	1	3
Technical failure	2	1
Loosening	1	1
Missing	2	1

Overall, the revision rate due to infection was 1.8% (5 revisions) among the patients with a secondary arthroplasty and 0.7% (4 revisions) among patients with a primary arthroplasty. The number of patients is too small for meaningful statistical comparison.

**Figure 2.**
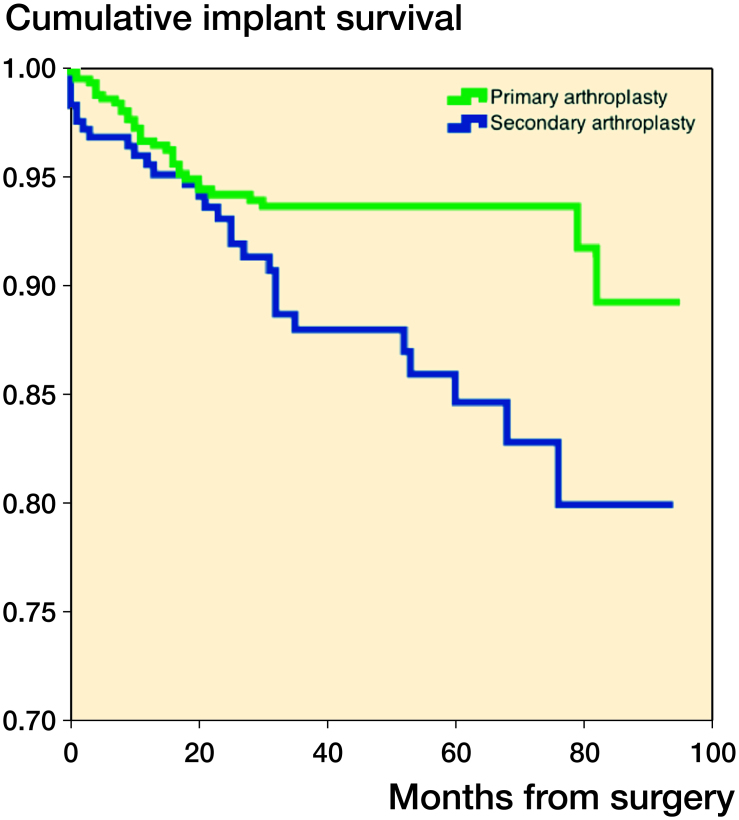
Implant survival functions of primary arthroplasty (green) and arthroplasty after failed osteosynthesis (blue).

The mean time to revision in the group of patients with a shoulder arthroplasty after a primary failed osteosynthesis was 59 (27) months and 53 (24) months in the group of patients with a primary shoulder arthroplasty (p = 0.4) (Figure 2). The relative risk of revision was 2.0 (95% CI 1.2–3.2) for an arthroplasty after failed osteosynthesis with a primary shoulder arthroplasty as reference.

## Subgroup: locking plate

**Figure 3.**
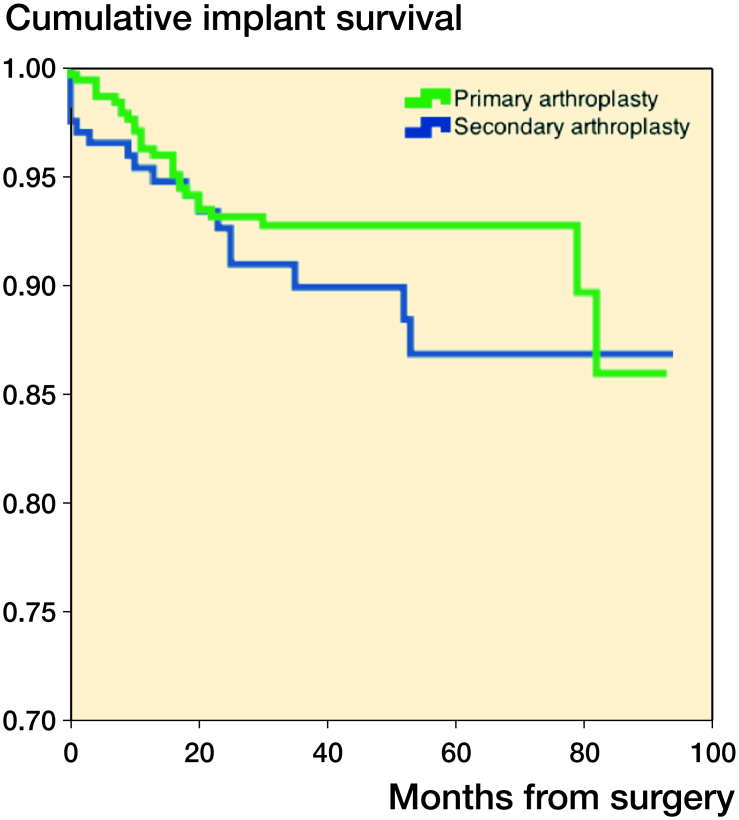
Implant survival functions of primary arthroplasty (green) and arthroplasty after failed locking plates (blue).

A locking plate was used in 208 (73%) of the patients with a primary osteosynthesis. The mean age in this subgroup was 66 (12) years and 155 (75%) patients were women. Avascular necrosis (39%) and fracture displacement (20%) were the major reasons for failure leading to shoulder arthroplasty (Table 4). The mean WOOS of patients with a shoulder arthroplasty after failed locking plate was 46 (24). The relative risk of revision was 1.5 (95% CI 0.8–2.6) for an arthroplasty after a failed locking plate with a primary shoulder arthroplasty as reference (Figure 3).

**Table 4.** Mode of failure of locking plates and outcome

		Mean	Revisions
Mode	n (%)	WOOS	n (%)
Avascular necrosis	82 (39)	43	6 (33)
Fracture displacement	41 (20)	42	6 (33)
Pseudarthrosis/non-union	27 (13)	53	4 (22)
Secondary arthrosis	4 (2)	22	0 (0)
Infection	5 (2)	30	1 (6)
Screw penetration	8 (4)	45	0 (0)
Pain	8 (4)	30	1 (6)
Rotator cuff tear	4 (2)	52	0 (0)
Implant failure	6 (3)	46	0 (0)
New fracture	0 (0)	–	0 (0)
Missing	23 (11)	47	0 (0)

## Discussion

We found a difference in WOOS of 6 points and a relative risk of revision of 2.0 for an arthroplasty after failed osteosynthesis with a primary shoulder arthroplasty as reference.

No value for minimal important change (MIC) regarding WOOS score in patients treated with shoulder arthroplasty has been reported. A study determining the MIC of 4 shoulder-specific PROMs (Simple Shoulder Test, Disabilities of the Arm, Shoulder and Hand (DASH and Quick DASH), and the Oxford Shoulder Score) found MIC values of 2.2, 12, 13, and 6 respectively (van Kampen et al. 2013), equivalent to 10–18% of the total score. Therefore, it is questionable whether the difference of 6 points in WOOS should be regarded as clinically relevant.

A locking plate was the most common type of osteosynthesis (73%). The subgroup of patients treated with this kind of osteosynthesis was comparable to patients treated by all types of osteosynthesis, regarding demographics and patient-reported outcome. However, the risk of revision among the patients treated with shoulder arthroplasty after failed locking plate was 1.5, indicating a lower revision rate among patients treated with a locking plate, compared with osteosynthesis in general.

The response rate of WOOS was 77% for those patients who were available for follow-up. A systematic review of postal self-administrated questionnaires used in health research reported an average response rate of 65% (Nakash et al. 2006). The Danish version of WOOS has been translated and cross-culturally adapted (Guillemin et al. 1993) and validated using classical test theory (Rasmussen et al. 2013), but only for patients with osteoarthritis treated with a shoulder arthroplasty.

When considering a revision arthroplasty several factors may influence the decision-making process, including patient age, co-morbidity, functional level, experience of the surgeon, the resources available, and the ability of the implant to be revised with an adequate outcome (Rasmussen et al. 2012). Thus, the indication for revision arthroplasty is not fully known and revision rates do not necessarily reflect clinical outcome.

The risk and burdens of additional surgery should be accounted for when deciding on the primary surgical procedure. Several studies have analyzed possible risk factors for failure of osteosynthesis of proximal humeral fractures. Petrigliano et al. (2014) reported an age between 50 and 64 as the only significant factor for revision arthroplasty after primary osteosynthesis, whereas Hardeman et al. (2012) reported AO type C fractures as the only significant risk factor. Spross et al. (2012) reported that heavy smokers and fracture-dislocations had a significant increased risk of complications.

We used a matched group of patients with a primary shoulder arthroplasty to have a reasonable understanding of what surgeons can expect after a primary shoulder arthroplasty. However, the groups were not matched by co-morbidity, arthroplasty type, fracture complexity, or other factors with a possible influence on outcome. Furthermore, the group of patients with a secondary shoulder arthroplasty represents only patients with failure after primary surgery. This could further contribute to the risk for poor outcome if the 2 groups are not comparable biologically, or they may have a different injury pattern. However, we were unable to correct for this in our study design.

We only report postoperative outcome. Thus, we are not able to report any change in outcome related to the arthroplasty procedure. Only a few small studies have reported the outcome after secondary treatment of primary failed osteosynthesis of a proximal humeral fracture with a shoulder arthroplasty. Hussey et al. (2015), Dezfuli et al. (2016), and Grubhofer et al. (2017) only reported reoperations after primary failed osteosyntheses with a reverse shoulder arthroplasty. We included reoperations with all kind of prostheses, but a subgroup analysis showed an average WOOS of 46 for both stemmed hemiarthroplasty and reverse shoulder arthroplasty. In the studies by Hussey et al. (2015), Dezfuli et al. (2016), and Grubhofer et al. (2017) the clinical outcome was reported to be acceptable, but it was not possible for them to report the risk of revision because of small sample size (11–44 patients) compared with the 285 patients included in our study. Our large number of patients enabled a separate analysis on reasons for osteosynthesis failure. It seems likely patients who had a revision because of an infection had a markedly worse mean WOOS at 32 points and among these patients no less than one-third had a new revision arthroplasty; however, the number of patients was too small for a meaningful statistical comparison.

Finally, it is important to stress that this study does not report or discuss the results of either osteosynthesis or other treatment modalities of failed osteosynthesis in general.

In summary, compared with primary arthroplasty for proximal humeral fracture, we found an inferior patient-reported outcome and a substantial risk of revision for patients treated with a shoulder arthroplasty after failed osteosynthesis of a proximal humeral fracture. The risk and burdens of additional surgery should be accounted for when deciding on the primary surgical procedure. More knowledge on patient-reported outcome after non-revised osteosynthesis and nonsurgical approaches is needed to inform future evidence-based decision-making.

MK: study design, data collection, data analysis, writing of the draft paper, and revision of the paper. JR, SB: study design, data analysis, and revision of the paper. BO: study design and revision of the paper. BE, SJ: data collection and revision of the paper.

*Acta* thanks Carl Ekholm and Christian Gerber for help with peer review of this study.
